# Disentangling olfactory and visual information used by field foraging birds

**DOI:** 10.1002/ece3.4773

**Published:** 2018-12-11

**Authors:** Diana Rubene, Malin Leidefors, Velemir Ninkovic, Sönke Eggers, Matthew Low

**Affiliations:** ^1^ Department of Ecology Swedish University of Agricultural Sciences Uppsala Sweden; ^2^ Present address: Department of Crop Production Ecology Swedish University of Agricultural Sciences Uppsala Sweden

**Keywords:** bird foraging, bird olfaction, herbivore‐induced plant volatiles, methyl salicylate

## Abstract

Foraging strategies of birds can influence trophic plant–insect networks with impacts on primary plant production. Recent experiments show that some forest insectivorous birds can use herbivore‐induced plant volatiles (HIPVs) to locate herbivore‐infested trees, but it is unclear how birds combine or prioritize visual and olfactory information when making foraging decisions. Here, we investigated attraction of ground‐foraging birds to HIPVs and visible prey in short vegetation on farmland in a series of foraging choice experiments. Birds showed an initial preference for HIPVs when visual information was the same for all choice options (i.e., one experimental setup had all options with visible prey, another setup with hidden prey). However, if the alternatives within an experimental setup included visible prey (without HIPV) in competition with HIPV‐only, then birds preferred the visual option over HIPVs. Our results show that olfactory cues can play an important role in birds’ foraging choices when visual information contains little variation; however, visual cues are preferred when variation is present. This suggests certain aspects of bird foraging decisions in agricultural habitats are mediated by olfactory interaction mechanisms between birds and plants. We also found that birds from variety of dietary food guilds were attracted to HIPVs; hence, the ability of birds to use plant cues is probably more general than previously thought, and may influence the biological pest control potential of birds on farmland.

## 1 INTRODUCTION

Foraging strategies of birds can have large impacts on ecosystem functioning, because their widespread occurrence and high mobility enables them to track resource abundance in space and time (Whelan, Tomback, Kelly, & Johnson, 2016). In systems where birds prey on arthropods, their predatory behavior influences trophic interaction networks by shifting the balance between herbivorous and predatory arthropods (Mooney et al., 2010). In turn, bird foraging activity reduces herbivore damage to plants and thus indirectly increases plant biomass (Boesing, Nichols, & Metzger, 2017; Mäntylä, Klemola, & Laaksonen, 2011). Because of this relationship between higher trophic level predators and primary plant production, there is increasing interest in understanding the mechanisms that focus terrestrial bird foraging behavior. This interest comes not only from a desire for ecologists to better understand bird foraging strategies and decision‐making processes (Kelly & Marples, 2004; Lindstrom, 1999; Roper & Marples, 1997; Yang, Walther, & Weng, 2015), but importantly also from an applied perspective where such knowledge might be used to improve plant yields or control pest species in production systems (Martin, Reineking, Seo, & Steffan‐Dewenter, 2013; Mols & Visser, 2007).

Recent studies provide evidence of direct interactions between birds and plants, showing that some birds can use herbivore‐induced plant volatiles (HIPVs) to identify herbivore‐infested trees by olfaction (Amo, Jansen, Dam, Dicke, & Visser, 2013; Mäntylä et al., 2008; Mäntylä, Kleier, Kipper, & Hilker, 2017). HIPVs are known to induce chemical responses in undamaged plants (Baldwin, Halitschke, Paschold, Dahl, & Preston, 2006; Karban, Yang, & Edwards, 2014; Kessler & Baldwin, 2001), repel herbivores (Ninkovic, Ahmed, Glinwood, & Pettersson, 2003), and attract predatory arthropods (Dicke, Poecke, & Boer, 2003; James, 2005). Thus, because these volatiles constitute a reliable signal of herbivore, and thereby predatory arthropod, presence (Kessler & Heil, 2011), it is reasonable to assume that birds could use these signals to direct their foraging behavior. However, even if birds can detect plant volatiles, it is still unclear to what extent birds use these volatiles or other olfactory cues when foraging, as the small number of field studies have produced inconsistent results (Koski et al., 2015; Mäntylä et al., 2008; Mäntylä, Blande, & Klemola, 2014; Mrazova & Sam, 2017).

Empirical evidence on interactions between trees and birds feeding predominantly on insects or other arthropods suggests that communication of the plant's herbivore load, benefits both plant and bird (Boesing et al., 2017; Mäntylä et al., 2011) (although it is possible that if birds primarily forage on predatory arthropods this could negatively affect plant fitness, through mesopredator release (Crooks & Soulé, 1999), because herbivores can become far more abundant after being “released” from the control of their main predators). From this, one could expect that the strength of selection to utilize such information is stronger in strictly insectivorous species compared to birds with a less specialized diet (Amo, Dicke, & Visser, 2016). However, it is unclear to what extent omnivorous bird species and other dietary guilds utilize olfactory cues to find a large variety of food types.

For any species, the reliability of plant volatiles and other olfactory cues will compete with direct visual signals of prey presence, especially at close distances. The importance of vision versus olfaction in foraging has rarely been studied in terrestrial birds, with the exception of bird responses to aposematic prey (Lindstrom, 1999; Roper & Marples, 1997). Studies on Procellariiform seabirds (Nevitt, 2008) and the Oriental honey buzzard (*Pernis orientalis*) (Yang et al., 2015) indicate that birds use both vision and olfaction either hierarchically or in combination to identify foraging sites or preferred food sources. Great tits (*Parus major*), predominantly insectivorous during summer, can identify herbivore‐damaged trees without any prey cues by use of olfaction alone, but not vision alone; however, the effect of visible prey was not tested (Amo et al., 2013). Thus, it is likely that both vision and olfaction can be used by different bird taxa, but the relative importance of olfactory HIPV cues and visual prey presence have never been tested in birds foraging on arthropod prey in natural field conditions.

Despite previous experiments suggesting links between the production of HIPVs and bird behavior (Amo et al., 2013; Mäntylä et al., 2008), critical questions remain unanswered, notably: (a) how do birds combine visual and olfactory information when foraging, and under what conditions do they prioritize olfactory information; and (b) are behavioral responses to HIPVs confined to bird species that forage predominantly on herbivorous insect prey or do species with a more generalist diet or those feeding on predatory arthropods (which are only indirectly linked to the plants) also respond to HIPVs? To begin addressing these questions, we tested the attractiveness of HIPVs and the relative importance of olfactory cues and visual prey presence to wild birds in an agricultural field setting. We presented plant volatiles and visible prey together or separately to birds, and expected that visual information would be more important when both types of cues were present, while olfactory cues would guide bird foraging choices if no visual information was available. Our study was conducted in farmland dominated by cereal crops and pastures, and specifically targeted ground‐foraging bird species that are predominantly insectivorous during the breeding season. These species, particularly farmland birds utilizing crops as habitat, often search for their insect prey in dense vegetation where visual information may only be available at very short distances; thus they could expected to benefit from using plant olfactory cues. If these birds, particularly opportunistic foragers, were attracted to HIPVs, it would indicate a much broader and general use of plant cues by birds and have broad implications for the role of birds in trophic interaction networks.

## 2 METHODS

### 2.1 Study area

The study was conducted in agricultural landscapes in Uppland, south‐central Sweden, near Lövsta Research center (59°49′N, 17°48′E), Svista (60°6′E, 17°36′E) and Viksta (59°56′N, 17°35′E). The study landscapes were dominated by organic farming with cereals (mainly barley *Hordeum vulgare* and wheat *Triticum aestivum*), lay fields containing clover (*Trifolium* spp.) and pastures with natural grassland vegetation for cattle grazing, interspersed with small forest patches. The field experiment was carried out during July 2016 and May to August 2017.

### 2.2 Experimental setup

We selected sites with short and sparse vegetation at the edges of crop fields or grasslands, or in the margins between crops and forest edges; in the early season 2017 sites were characterized by bare ground and previous‐year grass and vegetation density increased during the season. To test birds’ preferences, we used a standardized setup with three petri dishes (ø 0.2 m) placed at the points of an equidistant triangle with 2 m sides. A wildlife motion detection camera (Scout Guard 880 MK, Scout Guard 550) was installed to overlook all three dishes. Up to nine experimental sites were set up simultaneously, 100–1,000 m apart. These were frequently moved to new locations (1–3 days) after being discovered by birds, to minimize the risk that birds learned the location and same individuals revisited the plots several times. In total, 45 experimental sites were established during the course of the study with a 3‐dish choice setup in each, but many of these were never visited by birds and the final data came from 20 sites where we had bird data. We did not observe any patterns in visitation which we could attribute to habitat type, and we did not attempt to evaluate this as the habitat surrounding the experimental sites was similar, for example, all sites were in or close to grasslands (typically on the crop/grassland border).

We used three experimental designs to test birds’ preferences for olfactory and visual cues. From these three experimental setups, we expected to better determine how birds react in a natural foraging situation to the presence of HIPV cues when prey were visible or hidden, and how birds decide where to investigate first when they can chose between cues (i.e., visual vs. olfaction). Two of these designs held the visual information constant while varying the odor cues between the dishes, while the third design separated the visual and odor cues from each other. Experimental design 1 (“visible prey”) contained insect prey (2 dead field crickets and 5 mealworms) in all three dishes placed on top of a layer of saw‐dust. The odor was varied between dishes so that one dish contained HIPV (see below), the second contained natural lemon oil (positive odor control) and the third had no odor (control). In experimental design 2 (“hidden prey”), the prey in all dishes were hidden under the saw‐dust, while the olfactory cues were the same as in design 1. The third experimental setup (“odor‐visual separation”) was designed to provide more information about how birds prioritized visual versus olfactory information by completely separating these cues; one dish contained HIPVs (odor only), another contained plastic spiders to eliminate any prey odor (visual only), and the third dish contained no cues (control). The position of dishes with different treatments was rotated in relation to the position of the camera between experimental sites to eliminate any effect of the camera itself. We expected the potential preference for HIPVs to be highest in the “hidden prey” setup, as birds may rely to a larger extent on plant cues when no visual cues are present.

### 2.3 Olfactory cues and arthropod prey

We used synthetic methyl salicylate (MeSA) as HIPV treatment. MeSA is an aromatic compound naturally produced by plants under attack from phloem‐feeding herbivores like aphids (Staudt et al., 2010). The role of MeSA in plant–plant and plant–insect interactions has been demonstrated in cereal plant species (Ninkovic et al., 2003) as well as other plant systems (James, 2005; Orre, Wratten, Jonsson, & Hale, 2010; Snoeren et al., 2010; Tang, Zhao, & Gao, 2013). MeSA is a typical induced compound naturally found in our study habitat, as aphids are the main herbivorous pests of cereal crops. Additionally, it is known to attract several taxa of predatory and parasitoid arthropods which feed on aphids (James, 2005; Simpson et al., 2011; Zhu & Park, 2005). Thus, we expected that birds foraging in farmland habitats would have encountered MeSA, and that it had a potential to indicate presence of arthropod prey which many farmland birds feed on. For field application, we incorporated MeSA in circular white wax pellets (ø 3 mm), which have a stable release rate and produce biologically relevant MeSA concentrations, similar to those released by aphid host plants (Ninkovic et al., 2003). The wax pellets contained 10% MeSA and were produced at the department of ecology at the Swedish University of Agricultural Sciences in Uppsala, by mixing waxes CPW400 (Trecora, USA) and MicroVax LMP (Shell, Netherlands) in proportion 65:35. The details of the technical procedure have been described in Ref. (Ninkovic et al., 2003). A positive odor control (lemon oil) was used to examine the possibility that birds may simply respond to odor, or that their response was due to multi‐modal cues being present in HIPV+prey treatment. While we could not rule out some volatiles in lemon oil potentially mimicking the HIPVs we were testing, this would only become an interpretation issue if the birds chose the lemon oil treatment at levels similar to those of the HIPV treatment. Further, potential concerns about lemon having a repellent effect would become an issue only if we observed a lower choice frequency for lemon odor compared to control. Many aromatic plants are repellent to insects, but are nevertheless used as stimuli in behavioral experiments with birds (Mennerat, Bonadonna, Perret, & Lambrechts, 2005). Finally, we cannot account for or compare the relative strengths of HIPV and lemon odor, since the physiology/neurology of how birds perceive different odors is so far unknown. We assume that the MeSA and lemon odors were perceivable by the birds as we could ourselves perceive both odors from the dish, but we presume that the HIPV odor was more constant over time while lemon oil evaporated faster and thus varied more in relative strength.

Field crickets and mealworms were purchased alive from a pet store, euthanized by cooling and then freezing for at least 24 hr, and only taken out just before use. If crickets were not eaten after 3–4 days, they were replaced. The wax pellets and lemon oil were replaced every time a setup was moved to a new location (i.e., every 1–3 days), after heavy rain or if the dish had been moved by birds. We used crickets and mealworms as prey, because they represent the common prey types of ground‐foraging farmland birds, in terms of size range and taxa. For the artificial prey, we used black plastic spiders similar in size to the field crickets and also to wolf spiders (Lycosidae), which are among the most common predatory arthropods in the study habitat and are known to feed on cereal aphids (Roubinet et al., 2017).

### 2.4 Data recording

The cameras were set to record a 15 s long video sequence when birds came within the limits of the infrared photo sensor. From the videos, we recorded the first dish inspected by each individual. We set the following criteria for a “choice” to be recorded: bird standing by dish with beak crossing threshold of the dish edge, or tilting head as to inspect dish, picking in dish, or walking over the dish or past the dish close enough to touch it (from the resolution of the videos it was not always possible to determine if the bird touched the dish). As the identity of each bird could not be determined, we used a reset time of 40 min, which means that any visiting bird of the same species within this time frame was assumed to be the same individual and no additional data were collected.

Due to the difficulty to identify individual birds and the relatively short recording time, we did not estimate visitation rate to all dishes. Generally, the birds either revisited all dishes in a systematic manner until all prey had been found (corvids) or approached only one dish and then left (most other species). Thus, visitation rate would not be a very informative measure for our experimental setup. Most bird species/individuals found and consumed the prey in the visible and hidden prey setups; however, a few individuals inspected the dish without consuming the prey. This was observed for the visible prey as well as for the hidden prey setups, so it was unlikely due to the type of information the birds received (visual vs. olfactory), but probably due to different levels of neophobia.

The camera only captured a limited area surrounding the dishes, making it unfeasible to assess the direction from which birds detected or initially approached the setups before making a choice. Dishes were placed relatively close together to make sure the whole setup was discovered simultaneously and, until there is more research done on the scale/distance at which birds perceive odors, the importance of distance to each dish relative to e.g., the landing position of a bird cannot be evaluated.

### 2.5 Analysis

Our aim was to test if the birds preferred the MeSA treated dishes within each experimental setup, and if bird preference differed depending on potential prey visibility. We used a multinomial distribution to estimate the probability of each of the three available options being approached first when a bird visited an experimental setup. We separately modeled these probabilities for each of the experimental designs (visible, hidden and odor‐visual separation) in addition to a general model examining first choice probability for the two experiments that held the visual information constant between choices (visible and hidden prey). This allowed us to compare first choice estimates of MeSA versus non‐MeSA when prey were present and visible, present and not visible, present (regardless of visibility) and not present. From these comparisons, we could disentangle the relative effects of MeSA on foraging decisions relative to visible prey. To best determine the magnitude and probability of a difference between these different treatments and groups, we used a Bayesian framework which allowed us to generate posterior probability distributions for each variable of interest and their differences. To account for the possibility that the same bird visited an experimental setup multiple times and biased the recordings at that site, records of the same bird species in the same location and setup were treated as the number of trials (N) in the multinomial likelihood. We implemented our analyses in JAGS (Plummer, 2003) called from R (R Core Team, 2017) using the “rjags” package (Plummer, 2016). We assumed an equal probability of choice for the prior (Dirichlet (1,1,1)) and used a cumulative distribution function (ecdf) to calculate probabilities that the preference for the treatments differed from each other. The ecdf represents the probability that one group is larger than another (i.e., these are calculated as the difference between the choice groups, with a probability of 0.5 indicating that this difference is centered on zero and has no effect, with increasing probabilities demonstrating greater certainty that there is a difference between treatments). Thus, probabilities of the direction and magnitude of between‐group differences are presented.

## 3 RESULTS

**Figure 1:**
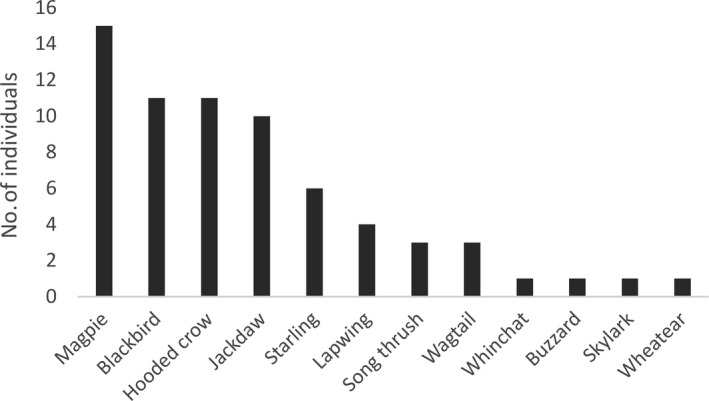
Species of birds observed in the study and number of individuals of each species: magpie *Pica pica*, blackbird *Turdus merula*, hooded crow *Corvus cornix*, jackdaw *Corvus monedula*, starling* Sturnus vulgaris*, lapwing* Vanellus vanellus, *song thrush *T. philomelos, *white wagtail *Motacilla alba, *whinchat *Saxicola rubetra, *buzzard* Buteo buteo,* skylark* Alauda arvensis,* and wheatear *Oenanthe oenanthe*

**Figure 2:**
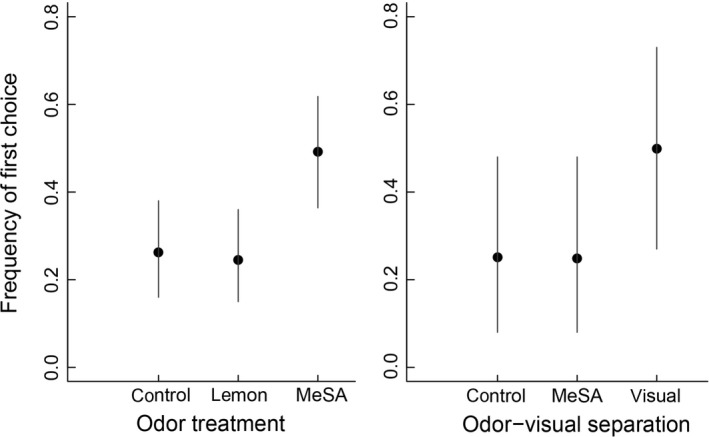
Estimated frequency of foraging first choice for field experimental setups with visual information held constant and odor treatments varied (visible +hidden prey setups; left panel), and where odor (MeSA) and visual information were completely separated (right panel). Estimates are means with 95% Bayesian credible intervals. For separate estimates of the visible and hidden prey designs comprising the left panel, as well as between‐group differences, see Table 1

Twelve bird species were recorded during the experiments, with most of the visits from corvids *Corvidae* (36 individuals), thrushes *Turdidae* (15 individuals), and starlings *Sturnus vulgaris* (6 individuals) (Figure 1). There was a clear choice demonstrated in the experimental plots for 20 birds visiting the visible prey setup, 34 birds visiting the hidden prey setup, and 13 birds visiting the odor‐visual separation setup. When visual information was the same across the three choices within an experimental setup (visible and hidden prey designs, *N* = 54), there was a clear preference for the MeSA treated dish (Table 1; Figure 2). This effect was independent of whether the prey were visible or hidden (Table 1). However, when the choices involved different visual information (odor‐visual separation design, *N* = 13), birds ignored the MeSA olfactory cue and prioritized the visual information instead (Table 1; Figure 2).

**Table 1:** Estimates for the probability of first choice in the three experimental designs (visible prey, *n* = 20; hidden prey, *n* = 34; and odor‐visual separation, *n* = 13) and the combination of the visible and hidden prey experimental designs. In addition, the between‐treatment differences are shown for each experimental design, and the probability that these differences are greater than 0 (Probability A > B)

Treatment estimates	Difference between‐group estimates		
	Group comparisons	Mean ± *SD*	Probability A > B
Odor variation (visible prey)			
MeSA = 0.52 ± 0.10	MeSA > control	0.26 ± 0.17	0.93
Lemon = 0.22 ± 0.08	MeSA > lemon	0.31 ± 0.16	0.96
Control = 0.26 ± 0.09	Control > lemon	0.04 ± 0.14	0.62
Odor variation (hidden prey)			
MeSA = 0.46 ± 0.08	MeSA > control	0.19 ± 0.13	0.91
Lemon = 0.27 ± 0.07	MeSA > lemon	0.19 ± 0.13	0.92
Control = 0.27 ± 0.07	Control > lemon	0.00 ± 0.11	0.50
Odor variation (1 & 2 combined)			
MeSA = 0.49 ± 0.06	MeSA > control	0.23 ± 0.11	0.98
Lemon = 0.25 ± 0.05	MeSA > lemon	0.25 ± 0.11	0.98
Control = 0.26 ± 0.06	Control > lemon	0.02 ± 0.09	0.59
Odor‐visual separation			
Visual = 0.50 ± 0.12	Visual > MeSA	0.25 ± 0.20	0.88
MeSA = 0.25 ± 0.10	Visual > control	0.25 ± 0.20	0.89
Control = 0.25 ± 0.10	MeSA > control	0.01 ± 0.17	0.51

Note

All estimates are the means and standard deviations derived from the Bayesian posterior distributions of the multinomial analyses.

## 4 DISCUSSION

Our results indicate that birds are attracted to herbivore‐induced plant volatiles (HIPVs) in agricultural habitats, and that they use HIPV cues under certain circumstances when making foraging decisions. The different experimental designs demonstrated that birds prioritize visual information when visual and olfactory cues are separated, but when visual information is held constant they use HIPVs to guide their foraging choices. The effect of HIPV‐directed behavior when prey are hidden is similar to studies showing that insectivorous birds are more likely to visit herbivore‐infested trees, even if they do not see the herbivores themselves (Amo et al., 2013; Mäntylä et al., 2008). Somewhat surprisingly, birds showed the same preference for HIPVs to guide their initial foraging choice even when insect prey were visible. This is the first study which addresses importance of HIPVs when visible insect prey are present. Under such conditions, birds may still choose a foraging location based on signals from plants, because without any variation in visual information HIPVs may indicate a richer local food source or provide additional cues by, for example, affecting camouflage of prey against plant background (Koski et al., 2017) or perhaps indicating suitable foraging habitat where they are likely to find prey. Similarly in the Oriental honey buzzard, it prefers food containing olfactory pollen cues when offered visually identical options (Yang et al., 2015). In both studies, it was important that the birds could not assess the difference between options based on visual information alone. This could be seen in the odor‐visual separation setup where the preference for MeSA was lost when birds could choose between visible prey with no odor and an odor with no prey.

To date, several studies have tried to identify specific chemical compounds which may be used by birds to identify herbivore‐infested plants (Amo et al., 2013; Mäntylä et al., 2008, 2014, 2017) and suggested that birds might smell, for example, α‐pinene, α‐farnesene, or linalool, which were produced in higher amounts in trees and shrubs during herbivore attack (Amo et al., 2013; Mäntylä et al., 2008, 2014, 2017; Mrazova & Sam, 2017). However, most studies attempting to apply a single compound or an artificially produced blend have failed to attract birds in nature (Koski et al., 2015; Mäntylä et al., 2014), but see (Mrazova & Sam, 2017) who used methyl jasmonate: MeJA; while those using real herbivore infestations have been more successful in demonstrating an effect (Mäntylä et al., 2008, 2014). We used a single volatile compound, MeSA, which is known to function in plant–plant and plant–insect communication (Dicke et al., 2003; Ninkovic et al., 2003). Even though MeSA was placed inside petri dishes and not in direct contact with vegetation, it is possible that it induced a response in surrounding plants, which then may have released a broader volatile blend. Thus, we cannot determine if the birds in our study responded to MeSA alone or to additional compounds produced by the surrounding vegetation. MeSA is not among the compounds previously identified by other studies linking HIPVs and birds, but is an important signal substance in systems with aphid herbivores like grasses and cereals (Ninkovic et al., 2003), which was the dominant vegetation type in our study sites. Thus, given the widespread occurrence of birds in various habitats dominated by different plant species, it seems unlikely that birds are tuned to detect one or a few specific compounds. It is more likely that their response to olfactory plant cues is flexible and driven by associative learning from foraging experience in specific habitats, which in also known to be the main mechanism driving the responses of arthropod predators and parasitoids to HIPVs (Kessler & Heil, 2011).

A large body of evidence on insect predators and parasitoids shows that they can learn to associate a wide range of plant volatiles with their herbivorous prey, despite a weak innate preferences in naïve predators (Kessler & Heil, 2011). Similarly, learning is very important for birds’ ability to use olfactory foraging cues, as (Amo et al., 2013) showed that insectivorous birds orientate toward plant volatiles if they have been previously exposed to these volatiles in combination with food, but the same is not observed for naïve birds (Amo et al., 2016). Thus, it is likely that the preferences we observed result from previous foraging experience. The birds in this study could not have learned to associate MeSA with food during the course of the study, that is, if same individuals approached the experiment repeatedly, because MeSA was not combined with higher food abundance compared to other treatments. Thus, if anything, our setup was more likely to train birds to ignore the MeSA cue as it did not contain any additional reward.

In both the visible and hidden prey treatments, there was likely some prey odor present as both crickets and mealworms have a noticeable smell. While prey odor cannot account for the observed results (since all dishes had the same prey concentration in them), it could have been important for birds’ interest to approach the experiment as we obtained a higher encounter rate for setups containing real prey compared to the artificial prey. Thus, the combined prey odor from all three dishes could have influenced bird attraction to the site, with the HIPVs then directing birds to an individual dish. This suggests that different cues may be used when choosing a foraging site versus making a choice once at the site, with odor potentially playing a role at both levels of decision‐making. Thus, the importance of olfaction and vision for selecting foraging sites at different scales still needs to be further explored.

In contrast to previous studies that have investigated bird attraction to plant volatiles, the bird species observed in this study were mainly omnivorous corvids (magpie *Pica pica*, hooded crow *Corvus cornix*, jackdaw *Corvus monedula*; Figure 1), often described as opportunistic species lacking a predominant diet. In addition, at least five species from different taxonomic and dietary groups including omnivorous common blackbird (*Turdus merula*), common starling (*Sturnus vulgaris*) and lapwing (*Vanellus vanellus*), and predominantly insectivorous white wagtail (*Motacilla alba*) and wheatear (*Oenanthe oenanthe*) were observed (Figure 1). Even though we did not have large enough sample size to analyze the responses on per species basis in the current study, our findings indicate that the ability to use plant cues is probably more general and widespread among birds than previously shown.

Empirical evidence showing mutual benefits of interactions between birds and plants has been collected from systems where interactions occur between trees and canopy‐feeding insectivorous bird species (Boesing et al., 2017; Mäntylä et al., 2011). We focused this study on a system where birds forage on the ground in cereal crops and grassland vegetation. In such systems, the main herbivorous pests are small (e.g., aphids) and birds are therefore more likely to forage on predatory arthropods rather than the herbivores themselves (Grass, Lehmann, Thies, & Tscharntke, 2017; Smith et al., 2009). These bird predators could exploit the plants “cry for help” to locate predatory arthropods, and by consuming them reduce herbivore suppression, hence the benefits to plants would be lost in accordance with the mesopredator release hypothesis (Crooks & Soulé, 1999). In fact, bird exclusion studies from systems with aphid pests show that bird foraging activity disrupts biological pest control and thereby affects plants negatively (Grass et al., 2017; Martin, Reineking, Seo, & Steffan‐Dewenter, 2015). Our study suggests that these negative effects may to some extent be mediated by birds’ use of plant volatiles.

## CONFLICT OF INTEREST

The authors have no conflict of interest to declare.

## AUTHOR CONTRIBUTIONS

DR designed and planned the study, carried out field work, participated in data analysis and drafted the manuscript; MLeidefors participated in planning of the study and carried out field work; VN and SE conceived the idea of the study and revised the manuscript; MLow carried out the data analyses and helped draft the manuscript. All authors gave final approval for publication.

## DATA ACCESSIBILITY

The data supporting results of this article are available from the Dryad Digital Repository, https://doi.org/10.5061/dryad.jv1n4sq.
